# Improving team-based care for children: shared well child care involving family practice nurses

**DOI:** 10.1017/S1463423617000160

**Published:** 2017-06-05

**Authors:** Grace Warmels, Sharon Johnston, Jolanda Turley

**Affiliations:** 1 MD Candidate 2017, University of Ottawa, Ottawa, ON, Canada; 2 Associate Professor, Department of Family Medicine, University of Ottawa, Ottawa, ON, Canada; 3 CCFP, Assistant Professor, Department of Family Medicine, University of Ottawa, Ottawa, ON, Canada

**Keywords:** inter-professional practice, Rourke Baby Record, Well child care

## Abstract

**Introduction:** Well child care (WCC) is the provision of routine preventative care and vaccinations to infants and children. In Canada, physicians provide the majority of this type of care, whereas in other developed countries, nurses provide most WCC. New models of shared care between nurses and family physicians should be explored. **Objective:** This pilot project aimed to evaluate the feasibility and acceptability of shared nurse–physician WCC for a cohort of healthy children. **Methods:** A total of 20 participants had nurse–physician alternating WCC visits, which were compared with physician-provided WCC visits. The feasibility was evaluated through chart audits and the acceptability was evaluated through interviews with the physicians, nurses, and the patients’ parents. **Results:** The results showed that physicians and nurses discuss a similar percentage of Rourke Baby Record topics, and that families and clinic staff were accepting of this new model of care. **Conclusion:** This intervention could liberate time for Canadian family physicians, thereby improving access to care.

## Introduction

Well child care (WCC) is the provision of routine preventative care and vaccinations to infants and children and, in Canada, the majority of this type of care is provided by family physicians and paediatricians (Guttmann *et al*., 2006; 2010). Some providers and payers are beginning to question the efficacy and efficiency of physician-provided WCC (Kuo *et al*., 2006; Tanner *et al*., 2009; Coker *et al.*, 2013). The role of nurses in WCC in several developed countries with strong primary healthcare systems, including Australia, the United Kingdom and the Netherlands, is well established (Condon, 2008; Schmied *et al.*, 2014; Benjamins *et al.*, 2015). A recent literature review, found that, in these three countries, WCC was mainly provided by nurses in a separate but parallel public health system designed specifically for this purpose (Turley *et al.*, 2017). Sometimes, there was little interaction or communication between the nurses and the children’s family physicians (Kuo *et al.*, 2009); however, this seems to be changing, especially in Australia and the United Kingdom (Hampshire *et al.*, 2001; Borrow *et al.*, 2011; Jeyendra *et al.*, 2013; Walsh and Mitchell, 2013). Trends in the studied countries included shifting WCC away from an authoritative medical model, with more focus on the biopsychosocial determinants of health and working in partnership with families, as well as an increased focus on higher-needs families with children at risk (Kruske *et al.*, 2006; Barbaro *et al.*, 2011; Wood *et al.*, 2013). The nursing role was well suited to this upstream approach to WCC, including screening and preventative care, health education, supporting families and linking with other allied health professionals and social services. The findings from this literature review informed the design of our proposed model of shared care for WCC.

Primary care in Canada and the United States is increasingly being provided by multi-disciplinary teams, which include registered nurses (Hutchison *et al.*, 2011). Family physicians need to explore optimal ways to collaborate and share responsibility for the ever-growing demands of primary care practice. WCC presents an opportunity to develop an effective and efficient model of shared care, maximising nursing scope of practice while maintaining the long-term patient–doctor relationship with parents and children. A shared-care model for WCC would decrease the time burden associated with caring for infants and children in the first two years of life and potentially enable family physicians to continue to accept infants into their practice, ensuring a continuously balanced practice spanning the age spectrum. This model also has the potential to liberate time for the family physician to care for more acutely ill patients.

This study aimed to evaluate the acceptability and feasibility of shared WCC between family physicians and practice nurses (registered nurses) for a cohort of healthy children at an academic inter-professional family medicine practice in Ottawa, Canada.

## Methods

This pilot study sought to develop a shared-care approach to WCC for family physicians and nurses, based on best evidence from the literature and input from the providers involved in its implementation. A mixed-methods, parallel convergent design was used combining qualitative and quantitative data gathering to generate a narrative description of outcomes. Recognising that this model involved changing the practice patterns of providers, we were guided in our approach by the theory of planned behaviour (Ajzen, 1991) and undertook extensive pre-implementation steps to address providers’ attitude towards the new model of care, their understanding of the group’s expectations, and their perception of their ability to carry out the tasks in the new model.

## Pre-implementation steps

### Ensuring the delivery of quality WCC

In order to delineate the practice we were aiming to target, we sought an established standard of WCC. In Canada, the Rourke Baby Record is endorsed by both the Canadian College of Family Physicians and the Canadian Pediatric Society as encompassing appropriate and comprehensive WCC (Rourke *et al.*, 2013).

The Rourke Baby Record is standardised, validated and evidence-based. It includes age-based recommendations for education and discussions related to nutrition, safety, parenting and behaviour, as well as physical exam manoeuvres, developmental screening, and pertinent growth parameters (Rourke and Leduc, 2016). The Rourke Baby Record is used as the standard of care for WCC provided at the study site, so it was adopted as such for the pilot project.

Furthermore, a competency mapping exercise was undertaken in order to ensure that the elements of the Rourke Baby Record were within the scope of practice of a Canadian registered nurse working in a primary care team. A review was done of the expected competencies of registered nurses (RNs) in Ontario, as reported by the provincial Nurses Association (Primary Care Task Force, 2012; College of Nurses of Ontario, 2014). These RN competencies were then mapped against the tasks listed in the Rourke Baby Record (ages two months to two years). This mapping exercise showed that the elements of WCC in the Rourke Baby Record were all within the scope of practice of a primary care RN in Ontario.

We then reviewed the Rourke Baby Record and expected care with the participating nurses who requested a refresher course to update their physical examination skills. Both nurses, despite their substantial experience working with children and infants in primary care, felt that there was a gap between their theoretical knowledge (based on training done many years ago) and their actual hands-on abilities to perform an accurate physical examination, which includes eliciting a red reflex of the eyes, for example. A 3 hour refresher course on examining infants was prepared and provided by the research team.

### Engaging the family physicians and nurses

The project was discussed at a team meeting and all of the physicians gave permission for nurses to see their patients at regular WCC visits. Two concerns were identified: (1) physicians felt that they would be missing out on enjoyable visits with families, and (2) physicians were concerned that nurse-provided WCC might lead to potentially weaker rapport with the children and their families which might undermine long-term or acute care provided to these patients. Based on the team’s feedback, it was decided that WCC visits would alternate between the nurse and family physician, rather than the nurses taking on all WCC visits in the study period. It was established that the nurses would begin providing WCC at two months, after the family physician had the chance to examine the baby and meet with the family several times to ensure appropriateness for the pilot project. Finally, it was agreed that children with ongoing medical problems or complex psychosocial issues, as identified by the patients’ family physicians, would not be appropriate for the pilot and would be excluded from recruitment.

### Intervention

Five physicians and two experienced primary care nurses were invited and consented to participate in this pilot study.

Patients, who met the inclusion criterion of aged two months to two years, were identified using a search of the clinic’s patient registry. Family physicians were provided their list of eligible patients and identified those on the list who met the additional inclusion criteria: infants born at term from an uncomplicated pregnancy, normal physical growth and development to date, no parental postpartum depression, and due for a WCC visit within the study period. Physicians could also recommend excluding patients based on medical or social complexity, poor follow-up, or other reasons they specified. In addition, as this study took place in an academic family practice unit, patients primarily cared for by family medicine residents were excluded, as this study may have interfered with an important component of the curriculum for these learners.

The parents were first approached by their own family physician to ask if they would be interested in a research assistant calling them about participation in the study. For those parents who expressed interest, the research assistant called them, explained the study and obtained verbal consent. For consenting participants, the subsequent WCC visits were booked alternating between the nurse and their own regular physician.

With the nurses’ input, it was decided that 30 min would be scheduled for each WCC visit, and the other practice nurse would cover the regular nursing duties during that time. The nurses always had a physician in clinic with whom they could consult at their discretion. Patients were also able to make follow-up appointments with their own physician if they desired. The nurses used the age-appropriate Rourke Baby Record, and documented the topics discussed and examinations performed during the visit in the patient’s chart. For the purposes of the chart audit, they also documented whether a physician or any other health professional was consulted, as well as the reason for the consult.

### Data collection

A chart review assessed which age-appropriate elements of the Rourke Baby Record were completed, the frequency of visits, and whether physician intervention was required for visits booked with the nurse, generating quantitative data. All of the charts of the study participants were reviewed, and for comparison, age-matched patients who had been seen by a physician for their WCC visit also had their chart audited.

Qualitative data came from semi-structured interviews with the participating nurses, and infant/children participants’ parents, as well as a focus group with the participating physicians.

### Analysis

The interviews and focus group were audio-recorded and transcribed. A coding template, developed from research questions, participating team member questions, themes from the literature, and emerging themes from the data during initial review was used to organise the data. The transcripts were read and coded by the two co-investigators (J.T. and S.J.). Thematic analysis was then conducted with emerging themes from the discussions.

Summary statistics from the chart audit were entered into Microsoft Excel and descriptive comparative tables were prepared.

## Results

**Figure 1:**
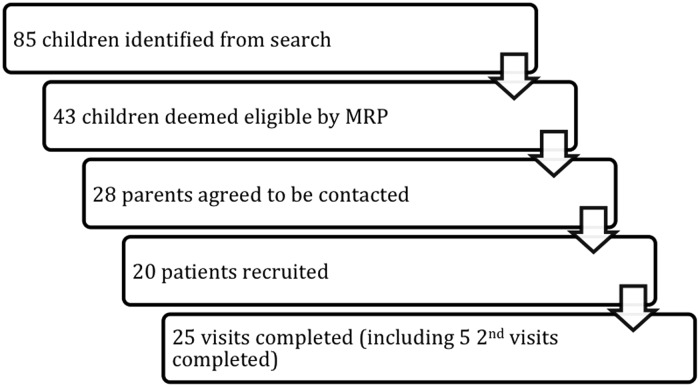
Study participation.

Figure 1 shows that 85 children were identified by the age criterion alone. A total of 43 children were deemed eligible to be contacted, with the most common reason for ineligibility being that the potential participants were being cared for on a regular basis by family medicine residents at the clinic. Of the 43 eligible children, 28 parents agreed to be contacted after their family physician spoke to them.

### Chart review results

#### Overall percentage of the Rourke completed

**Figure 2:**
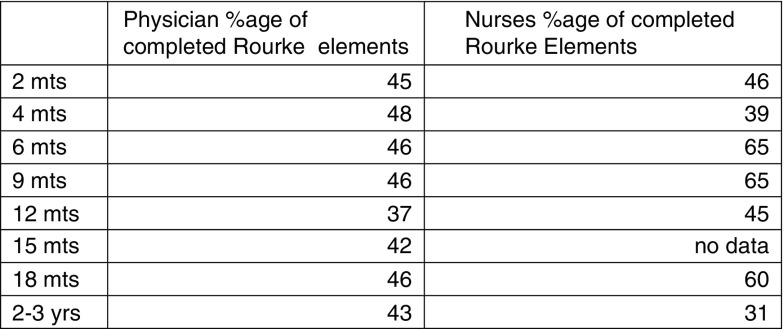
Percentage of completed Rourke elements.

**Figure 3:**
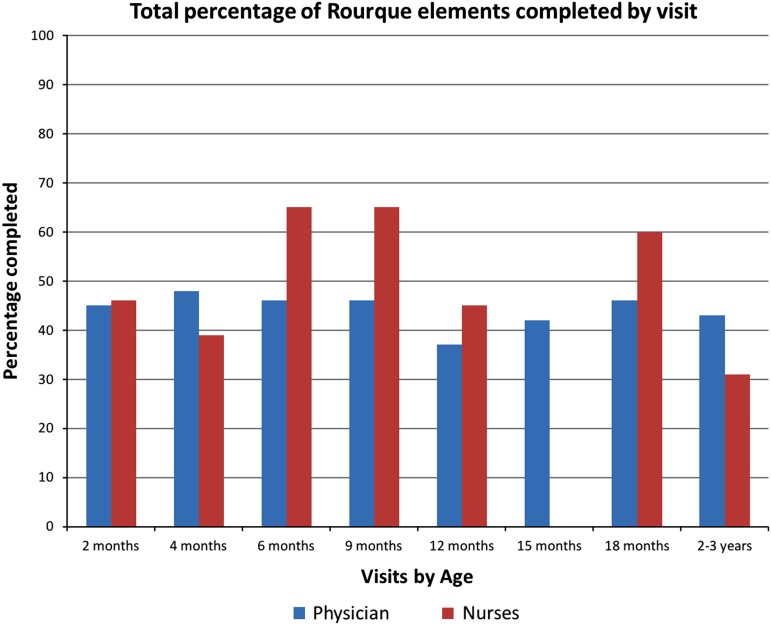
Percentage of Rourke elements completed by visit.

The chart review counted the number of Rourke Record items completed at each visit and found that nurses and physicians both completed a similar percentage of the Rourke Baby record items (see Figures 2 and 3). There was no nurse-provided 15-month visit within the time of intervention. It appears that, across all visits, nurses generally scored higher than physicians in the sections of environmental health and physical exam, whereas the other sections yielded comparable results between physicians and nurses. Of note, both physicians and nurses checked the patient’s growth parameters and developmental milestones 100% of the time at each visit.

#### Consults to physicians

Of the 25 visits, six resulted in consultation with a physician or nurse practitioner. Three of those visits included a physician coming to assess the patient immediately, the other three were seen in follow-up. Two patients were seen for a rash, another assessed for a possible head injury secondary to a fall. Others were referred to other services such as ophthalmology and orthopaedics or asked to see their family physician in follow-up for poor weight gain. One visit also included a referral for the patient’s parent to see a physician.

The vaccination rate was 100% for both the physician and nurse-provided WCC visits.

### Qualitative results: quotes and general themes

The post-visit phone interviews with parents revealed that all parents were generally satisfied with their well child check done by the nurse. Two parents stated that they would have preferred to still see a physician after the nursing visit, but three parents stated specifically that they preferred the nurse-only visit as it was efficient and complete. All parents answered that they would continue for their children to be patients at the clinic if this model of care became standard practice in the future. In response to this question, one parent stated:

‘I most certainly would. I think that it’s nice because I think it relieves some of the demands of the doctor, so I think we’re actually getting better service because of that. The way that we were dealt with is that if there were any concerns that the nurse didn’t feel she could address, she went and got the doctor. So, I thought it was very, very effective. I think it reduces the pressure on some of the doctors; and the use of the nurses, they are more than qualified in my opinion to be doing those checkups and to check those regular things. If there are questions that they don’t feel that they can address, as long as they’re comfortable to go to the physician, then I think it’s a fabulous way to move forward’.

The post-intervention interviews with the two participating nurses revealed that they both felt comfortable with the clinical aspect of providing WCC, but specifically because they felt that they had a supportive team of physicians for consultation if necessary. Both acknowledged the value of the ‘refresher course’ that had been offered by the research team but one nurse suggested that opportunities to practice and be observed doing some of the physical exam components would have been beneficial. Both nurses commented on the fact that they had a pre-existing relationship with the families and children and that this seemed to have facilitated the acceptance of this new model by the parents; they further commented that, if this model is implemented, they should see their own regular patients rather than covering someone else’s practice.

Both nurses felt that there could be logistical barriers to implementation of this model of care, in that they would need dedicated time to do the visits, without interruptions by the clerical staff or other providers, as happened frequently during the study visits. Both nurses felt that WCC falls well within the scope of practice of a primary care nurses and felt that the patients’ parents were open to the idea and satisfied with the visits. Both nurses felt they could easily work with a consulting physicians and efficiently address a patient question if needed.

‘I never felt like I was thrown in the jungle to do work without knowledge and support from the team’.

(Nurse 1)

‘I felt comfortable because of the team that I am working with’.

(Nurse 2)

Lastly, the physicians gave feedback in a focus group and the themes which emerged from this discussion were generally positive and supportive of the new model of care. A high degree of confidence and trust in the nurses’ skills and clinical judgement was stressed as a reason for the acceptability of this model. The physicians stated that they believed that the nurses had more time for the WCC visits and that they would be better at counselling. They speculated that patients might open up more to nurses about specific issues. The physicians all acknowledged that a shared office space and shared electronic medical records, with the capacity for chart-specific messaging, facilitated this shared-care model and encouraged communication. The physicians were somewhat concerned about losing continuity of care with their patients and preferred to be on site at the time of the nurses’ WCC visits so they would be available for consultation if the need arose. Lastly, certain specific billing issues were identified to be a possible barrier for assigning the 18-month visit to the nurses. The 18-month visit has relative importance for autism screening, and there is a lucrative billing fee that physicians would lose the opportunity to collect if the patient is seen exclusively by the nurse for this visit. Overall, however, all of the physicians felt that this model of care could be adopted in the future as standard practice for WCC visits in the study clinic.

## Discussion

This study’s goals were to test feasibility and acceptability of nurse-provided WCC in a family practice where the standard of care had been for all infants and children to be seen by a family physician at every visit. Most often, the nurse would have seen the child beforehand, measuring and weighing and giving vaccines, but a physician was always expected to complete the visit. Our pilot project was modelled on the type of care seen in many developed countries where registered nurses provide most WCC, and indeed, our competency mapping exercise showed that the tasks expected at a WCC visit are all within the scope of practice of a registered nurse in Ontario. Feedback from the nurses, family physicians and the patients’ parents revealed that all of them felt that this new model of care is an efficient use of time and resources. All participants agreed that the model was feasible within the academic family health team setting. This study also had five repeat WCC visits with the nurses, which is another strong indication that the families accepted this model.

Moreover, at the study site, the nurses and family physicians work alongside each other, using the same facilities and electronic medical records, making communication quick and easy and presenting to the families a cohesive, well-functioning team where the nurses could reach out to a physician without any barriers, if the need arose. The literature review undertaken in preparation for this pilot project found that in countries where WCC is provided away from the family physicians’ offices, nurses felt that communication was sometimes difficult (Barnes *et al*., 2004; Kuo *et al*., 2006; Turley *et al*., 2017). In both Australia and the United Kingdom, maternal and child care nurses are increasingly starting to work within family practices, in part to overcome this perceived barrier. The clinic where our study took place has taken advantage of co-location and direct communication methods to promote maximising nurses’ knowledge and skills to provide WCC. We are advocating the term ‘shared-care’ for this model as the nurses could provide the majority or all of the WCC, with the family physician as part of the team, available for direct or indirect consultation at any time.

Discussions with the physicians before the start of the study had identified concerns about potentially losing out on continuity of care with the children and their families but alternating the WCC visits between the nurse and the physician mitigated this issue. Moreover, both the physicians and nurses reported that having the child’s own physician available for consultation, if needed, would be preferable for the promotion of continuity of care. Lastly, having the WCC visit with the nurse already familiar with the family was also seen as a positive aspect of this study. One of the issues raised in the large study of WCC undertaken in the Netherlands was also the issue of continuity of care (ie, seeing the same nurse for most or all visits), and parents identified this as an important aspect of a positive experience (Benjamins *et al*., 2015). This will need to be taken into account and worked out by our team if this model of care is implemented in our clinic going forward.

In regards to the chart review, it was found that nurses generally completed a similar (or slightly higher) percentage of Rourke elements compared with the physician controls, however, both groups managed to complete only about 50% of the elements during the visits. This is very likely a reflection on the time limitation of WCC visits, and the large number of items listed for discussion in the Rourke Record. Many of the items listed could have been discussed once over the course of a number of visits (eg, fire-arm safety) and we do not currently have a method in our electronic medical records to delete items from the Rourke Record that would have been previously discussed, thereby over-estimating the number of items not covered. The chart review did not take into account whether a topic had been previously discussed with the family, so it was not possible to ascertain whether or not all of the Rourke elements were eventually discussed, albeit in different visits. However, the important aspects of WCC, including growth, developmental milestones and vaccines were covered 100% by both groups for all of the audited visits, reassuring us that our patients are receiving good preventative care.

We did not record the length of the nurse-provided WCC visits but the nurses had requested 30 min per visit and felt that this would suffice to provide the requisite care. Given more time, the nurses could discuss more of the Rourke elements, but further study would be needed to assess the efficiency gains of such a model for primary care teams and the impact of the re-allocation of work flow for team nurses.

The main limitation of this study was the small sample size. For this reason, our conclusions are descriptive and we could not make comparisons between WCC provided by the nurses and physicians. Another limitation was the lack of a 15-month WCC nursing visit.

## Conclusion

Based on this pilot project, shared WCC is a feasible and acceptable model of care for family practices in Ontario, where nurses work alongside physicians. This feasibility project successfully established a shared-care approach to WCC at a Family Health Team with good ‘buy-in’ from providers. It also established that completion of the Rourke Baby Record fits within the scope of practice of registered nurses. Both parents and healthcare providers generally found this model acceptable, and it was found to be feasible within the context of a highly collaborative primary care team.
